# Geographic characteristics of sable (*Martes zibellina*) distribution over time in Northeast China

**DOI:** 10.1002/ece3.2983

**Published:** 2017-04-25

**Authors:** Rui Zhang, Li Yang, Lin Ai, Qiuyuan Yang, Minhao Chen, Jingxi Li, Lei Yang, Xiaofeng Luan

**Affiliations:** ^1^ School of Nature ConservationBeijing Forestry UniversityBeijingChina; ^2^ School of ForestryBeijing Forestry UniversityBeijingChina

**Keywords:** climate change, conservation, historical change, *Martes zibellina*, potential distribution, sable

## Abstract

Understanding historical context can help clarify the ecological and biogeographic characteristics of species population changes. The sable (*Martes zibellina*) population has decreased dramatically in Northeast China since the l950s, and understanding the changes in its distribution over time is necessary to support conservation efforts. To achieve this goal, we integrated ecological niche modeling and historical records of sables to estimate the magnitude of change in their distribution over time. Our results revealed a 51.71% reduction in their distribution in 2000–2016 compared with the potential distribution in the 1950s. This reduction was related to climate change (Pearson's correlation: Bio1, −.962, *p* < .01; Bio2, −.962, *p* < .01; Bio5, .817, *p* < .05; Bio6, .847, *p* < .05) and human population size (−.956, *p* < .01). The sable population tended to migrate in different directions and elevations over time in different areas due to climate change: In the Greater Khingan Mountains, they moved northward and to lower elevations; in the Lesser Khingan Mountains, they moved northward; and in the Changbai Mountains, they move southward and to higher elevations. Active conservation strategies should be considered in locations where sable populations have migrated or may migrate to.

## 1 Introduction

The sable (*Martes zibellina*), a species of marten (family: Mustelidae), is a medium‐sized carnivore distributed widely in Russia, Mongolia, China, North Korea, and Japan (Proulx et al., 2005). It inhabits forests and is associated with coniferous taiga forests. In China, it is distributed in Northern Xinjiang and Northeast China (Monakhov, 2001b, 2005; Proulx et al., 2005). Due to commercial hunting and deforestation, the sable population has decreased dramatically in Northeast China since the l950s (1950–1959; Kashtanov et al., 2015; Monakhov, 2005, 2011a) and there is an increasing need for its conservation. Understanding changes in its distribution over time is necessary to support basic conservation efforts.

To understand the ecological and biogeographic characteristics of declining species populations, the current distribution should not only be examined but also placed within a historical context (Boshoff & Kerley, 2010; Davies, Colombo, Hanley, & Thompson, 2014; McClenachan, Ferretti, & Baum, 2012; Rick & Lockwood, 2012; Schipper et al., 2008; Turvey, Crees, & di Fonzo, 2015). Our previous research have highlighted multiple data resources that can be used to collect long‐term historical data, including gazetteers, journal articles, nature reserve surveys, and news (Yang et al., 2016; Zhang et al., 2016). In addition, previous research revealed that quantitative analyses are useful for assessing the utility and potential limitations of historical data to develop a roadmap for understanding the population size changes over time (Boshoff & Kerley, 2010; Channell & Lomolino, 2000; Davies et al., 2014; Hortal, Jimenez‐Valverde, Gomez, Lobo, & Baselga, 2008; Rick & Lockwood, 2012; Turvey et al., 2015; Yang et al., 2016; Zhang et al., 2016). However, finding complete datasets containing high‐quality information on the spatial distribution of biodiversity indicators within a region over time is difficult (Davies et al., 2014; Hortal et al., 2008). In practice, such historical data are often incomplete and spatially biased (Hortal et al., 2008; Yang et al., 2016; Zhang et al., 2016). Ecological niche modeling, which estimates the relationship between species records at sites and the environmental variables of those sites, can be a powerful tool to reconstruct historical distributions and reduce biases (Elith & Leathwick, 2009; Elith et al., 2011; Feng, Lin, Qiao, & Ji, 2015; Liu, Berry, Dawson, & Pearson, 2005; Phillips, Anderson, & Schapire, 2006). Maximum entropy modeling (MaxEnt) is considered to provide the best predictive performance among ecological niche models (Elith et al., 2011; Luo, Jiang, & Tang, 2015; Phillips et al., 2006; Zhang & Zhang, 2014). Therefore, we used MaxEnt to reconstruct the historical distribution of sable from historical records (Elith et al., 2011; Liu et al., 2005; Phillips et al., 2006).

We integrated ecological niche modeling with historical sable records to estimate the magnitude of change in their distribution over time. To achieve this, we (1) created a historical dataset of sable distribution from multiple resources (e.g., gazetteers and journal articles); (2) reconstructed climate data to represent the environmental variables; (3) estimated the potential habitat for each period using MaxEnt; and (4) evaluated changes in their distribution range and possible impact factors. Our results offer an opportunity to understand the ecological and biogeographic characteristics of species population decline, thereby improving the predictive power of conservation management for sables and other species in similar ecology niches.

## 2 Materials and Methods

### 2.1 Study area

Northeast China includes Heilongjiang Province, Jilin Province, and the northeast corner of Inner Mongolia (N40°5′–53°17′, E115°30′–135°06′; Yang et al., 2016; Zhang et al., 2016). The climate is a continental monsoon climate with a negative water balance. The annual precipitation is 400–1,000 mm, and the annual average temperature is 1–4°C, with a north–south temperature gradient of 25°C. The region includes coniferous forest, broadleaf mixed forest, secondary forest, woodland shrub and marshy grass areas, and contains more than 2,500 plant species, with a tree cover of ~42.9% (Xiaofeng et al., 2011; Yang & Xu, 2003). Coniferous forest is mainly located in the Greater Khingan Mountains, where it is characterized by *Larix gmelinii*. Broadleaf mixed forests are located in the Lesser Khingan Mountains, Changbai Mountains, and Wanda Mountains and are dominated by *L. gmelinii*,* Pinus koraiensis*, and *Betula platyphylla* (Yang & Xu, 2003). The forested area supports numerous wildlife species, including *M. zibellina* and its abundant prey (e.g., moles and hares).

**Figure 1:**
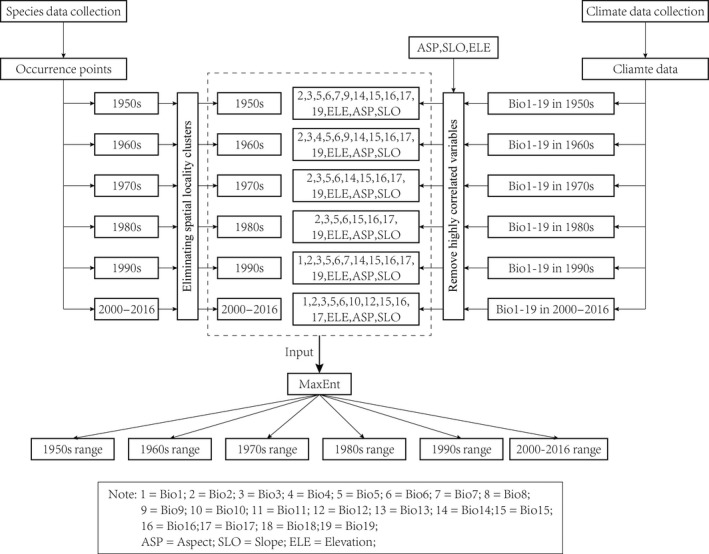
Approach for modeling species potential distributions

In this study, we obtained the boundary of Northeast China from the Thematic Database for Human‐Earth System (http://www.data.ac.cn/index.asp). The area covers 938,000 km^2^ with an elevation range of 2–2,576 m. The ecological data were obtained in a raster structure with a cell size of 1 km^2^ (Figure 1).

### 2.2 Data

Based on previous research (Yang et al., 2016; Zhang et al., 2016), we collected historical sable data from five sources: gazetteers, fauna records, nature reserve scientific surveys, scientific research, and news (see the Table S1 in Appendix S1). Conflicting records with unsubstantiated datasets, such as those lacking relevant or detailed descriptions, were excluded from the analysis. We recorded the locality information from all of the extracted records. In total, 1,028 records were collected. To eliminate potential biases caused by clustered occurrences, we removed duplicate records within the same cell. Ultimately, records were selected and divided into different time periods: 902 records in 1950s (1950–1959), 678 in 1960s (1960–1969), 489 in 1970s (1970–1979), 363 in 1980s (1980–1989), 147 in 1990s (1990–1999), 84 in 2000s (2000–2016).

Sables may be affected by human influences (Monakhov, 2001a, 2001b, 2011a, 2011b; Zhou et al., 2011), but the historical data of human activities (e.g., land cover, vegetation cover from the 1950s) are difficult to be compiled. The change of human population size can reflect the scale of urbanization, the deforestation, and the reclamation of farmland. Therefore, we collected human population size data from the first to the sixth national population censuses and several gazetteers (see the Fig. S2 in Appendix S3).

### 2.3 Environmental variable selection

Daily climate data were obtained from the surface daily climate dataset (code: SURF_CLI_CHN_MUL_DAY, ver. 3.0, http://data.cma.cn), including mean temperature, maximum temperature, minimum temperature, and precipitation (recorded daily at 20:00). Datasets from 1951 to 2010, from 72 meteorological stations, were downloaded. Environmental variables for 12 months per 10 years were calculated with ANUSPLIN ver. 4.36. This package facilitates the transparent analysis and interpolation of complex multivariate data using thin plate smoothing splines, which were suitable for this research. For each period, bioclimatic variables were derived from the monthly temperature and rainfall values to generate more biologically meaningful variables with the dismo package in R (R Development Core Team, Vienna, Austria). From this, we obtained 19 bioclimatic variables. Topography data were obtained from the SRTM 90 m Digital Elevation Database (http://srtm.csi.cgiar.org/). The slope and aspect data were calculated from the topography data. In total, 22 variables were obtained for further research.

Overfitting can result from highly correlated variables; therefore, we built a Pearson correlation matrix for 22 variables. We drew a network diagram (see the Table S3 in Appendix S2) using a raster structure with the igraph package in R to facilitate variable selection (Csardi & Nepusz, 2006; Hijmans et al., 2016). In the network diagram, highly correlated variables (|*r*| ≥ .8) were linked by lines and clustered together. We assumed that the climate variables could constrain the distribution during months when the species face a severe ecological environment in this region. Under the assumption that extreme climate index variables (e.g., BIO5, BIO6, BIO14, etc.) represented limiting factors for the species, we prioritized those variables among the correlated cluster. Each period had a unique set of variables for the ecological niche model (Figure 1; see the Table S3 in Appendix S2).

Eliminating spatial locality clusters can result in high correlations in models; therefore, we reduced the occurrence localities in each time periods within a specified Euclidian distance (10 km), according to topographic and climate heterogeneity, with the tool to spatially rarefy occurrence data in SDM toolbox ver. 1.1c (based on ArcGIS ver. 10.2; Brown & Anderson, 2014). Then, we analyzed the changes in sable distribution based on records in different periods (Table 1): 709 records in 1950s (1950–1959), 545 in 1960s (1960–1969), 403 in 1970s (1970–1979), 296 in 1980s (1980–1989), 124 in 1990s (1990–1999), 79 in 2000s (2000–2016).

**Table 1:** Estimates of range size and accuracy of species distribution models in different time periods in Northeast China

Period	Occurrence points	Records	AUC (Mean ± SD)	Range
1950s	902	709	0.714 ± 0.019	631,522
1960s	678	545	0.742 ± 0.018	607,354
1970s	489	403	0.773 ± 0.020	532,024
1980s	363	296	0.807 ± 0.023	447,252
1990s	147	124	0.845 ± 0.031	305,706
2000–2016	84	79	0.863 ± 0.033	304,932

Occurrence point data were collected from five sources and verified (see Section 2.2). Records were obtained from occurrence points with the tool to spatially rarefy occurrence data. All results were derived from the distribution models. Model accuracy was measured from the AUC curve (mean ± standard deviation [*SD*]). The ranges were determined as the total of the number of related pixels.

### 2.4 Species distribution modeling

We estimated the potential sable distribution in Northwest China over time using MaxEnt ver. 3.3.3k (Elith et al., 2011; Phillips et al., 2006). We randomly selected 80% of the occurrence records as calibrations to generate the model and used the other 20% as test data to evaluate the model. The model was developed with MaxEnt ver. 3.3.3k with the following parameters: number of random selected background points = 10,000; replicated run type = subsample; replicates = 20; auto feature; convergence threshold = 0.00001; and output format = logistic. The final model was the average of these replicates. To estimate its accuracy, we used the area under the curve (AUC) of the receiver operating characteristic to assess model fit (Feng et al., 2015; Phillips et al., 2006).

In order to transform our models from environmental suitability into presence–absence distributions, we used the threshold (*P*) calculated with the sensitivity–specificity sum maximization approach (Liu et al., 2005). We transformed logistic model output to a presence–absence map for each time periods using the mean threshold of maximum training sensitivity plus specificity logistic threshold as the cutoff. Then, all the outputs were divided into two groups: Outputs above the threshold (>*P*) were grouped as “present,” while all other values (<*P*) were considered to be “absent.”

### 2.5 Spatial analysis

It is important to quantify distribution change spatial patterns to gain a better understanding of threatening factors (Luo et al., 2015; Pacifici et al., 2015; Urban, Zarnetske, & Skelly, 2013). The predicted ranges for each period were determined as the total number of respective related pixels. To account for the complicated topography of Northeast China, we divided Northeast China into three regions: the Greater Khingan Mountains, the Lesser Khingan Mountains, and the Changbai Mountains. For each time period, the latitudes and longitudes of the range centroids and the elevation of the potential distribution were estimated (Barbet‐Massin et al., ). Then, the Pearson correlations between the change in distribution range and the change in latitude and longitude of the range centroids, as well as the elevation change of potential distribution, were estimated.

Human influence and climate are likely to be important factors affecting sable distribution (Kashtanov et al., 2015; Miyoshi & Higashi, 2004; Monakhov, 2001b, 2005, 2011a, 2011b; Proulx et al., 2005; Zhou et al., 2011). Therefore, we tested the Pearson correlations between range change and five factors: human population size, annual mean temperature (Bio1), maximum temperature of the warmest month (Bio5), minimum temperature of the coldest month (Bio6), and annual precipitation (Bio12).

The spatial analyses were conducted in ArcGIS (ver. 10.2; ESRI Inc., Redlands, CA, USA), Excel 2013 (Microsoft Corp., Redmond, WA, USA), and SPSS for Windows (ver. 20.0; SPSS Inc., Chicago, IL, USA).

## 3 Results

### 3.1 Model performance

The number of available records of sables in each decade ranged from 79 to 709 (Table 1). The average AUC for the consensus model was 0.791 ± 0.053 (Table 1). The period of 2000–2016 had the highest AUC among all periods, while the 1950s had the lowest.

### 3.2 Range change over time

**Figure 2:**
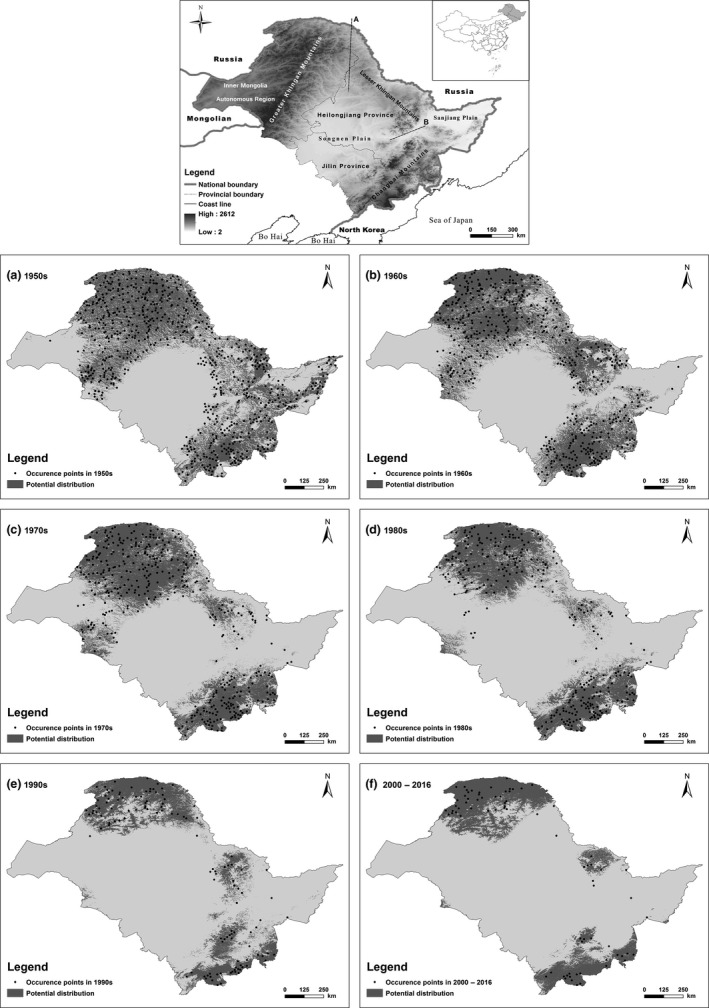
Potential sable distribution in different temporal periods (a‐f). The line in the study area indicates the boundary between two mountains. (A) Greater Khingan Mountains and Lesser Khingan Mountains; (B) Lesser Khingan Mountains and Changbai Mountains

Sables were widely distributed in all three areas of Northeast China, including the Greater Khingan Mountains, Lesser Khingan Mountains, and Changbai Mountains (Figure 2). In the 1950s, their potential distribution area was 67.3% of the total study area (938,000 km^2^). The potential sable distribution did not present obvious alterations until the 1970s. Compared with the potential distribution in the 1950s, the range decreased by 3.83% and 15.76% in the 1960s and 1970s, respectively. This decline worsened after the 1970s, with reductions of 29.18% and 51.59% in the 1980s and 1990s, respectively, compared with the 1950s. In 2000–2016, the distribution became concentrated in the core areas of the Greater Khingan Mountains, Lesser Khingan Mountains, and Changbai Mountains.

Understanding changes in geographic characteristics over time is important for conservation and environmental management. Our results suggest that the sable population in the Greater Khingan Mountains tended to move northward and to lower elevations (compared with the 1950s, range centroid shift, 147.90 km; elevation decrease, 63.55 m), while the population in the Lesser Khingan Mountains predominantly moved northward (range centroid shift, 66.55 km; elevation decrease, 23.91 m; Table 2; Table S5 in Appendix S3). Our study projected that the range shifted by only 67–195 km (0.96–2.79 km/year) in Northeast China over the past 70 years, which is lower than shifts estimated in other research (e.g., migrant birds in Afro‐Palaearctic, 5.56 km/year; ungulates in the Tibetan Plateau, 4.52 km/year; Mustelidae in the Western Hemisphere, 7.87 km/year; Levinsky et al., 2007; Luo et al., 2015; Schloss, Nunez, & Lawler, 2012).

**Table 2:** Pearson's correlation between sable range change with range centroids and elevation

Region	Versus range	Longitude	Latitude	Elevation
Greater Khingan Mountains	Pearson correlation	−.464	−.936	.876
	Sig. (two‐tailed)	.354	.006	.022
Lesser Khingan Mountains	Pearson correlation	.085	−.863	−.681
	Sig. (two‐tailed)	.873	.027	.137
Changbai Mountains	Pearson correlation	.517	.690	−.791
	Sig. (two‐tailed)	.294	.129	.061

### 3.3 Threatening factors

Global warming and human influences can force animals to alter their habitats, leading to habitat loss and fragmentation (Luo et al., 2015; Rands et al., 2010; Rondinini & Visconti, 2015; Willis, Gillson, & Knapp, 2007;). Compared with the temperature characteristics in the 1950s, the annual mean temperature, maximum temperature of the warmest month, and minimum temperature of the coldest month increased by 1.90, 1.29, and 4.41°C in 2000–2016, respectively (see Table S4 in Appendix S3). Meanwhile, annual precipitation was 13,686.39 mm greater in 2000–2016 than that in the 1950s. We assumed that human influence was associated with human population size. In 2010, the population of Northeast China (75.66 million) was nearly three times greater compared with that of the 1950s (27.18 million; see Fig. S2 in Appendix S3). Our results implied that sable range change was influenced by four factors, particularly annual mean temperature and human population sized (Table 3, specific information showed in Appendix S3).

**Table 3:** Pearson's correlation between sable range change with human population size and climate factors

Versus range	Pearson's correlation	Sig. (two‐tailed)
Human population size	−.956	.003
Annual mean temperature (Bio1)	−.962	.002
Maximum temperature of the warmest month (Bio5)	−.817	.047
Minimum temperature of the coldest month (Bio6)	−.847	.033
Annual precipitation (Bio12)	−.489	.325

## 4 Discussion

Our investigation using historical records to quantify the temporal and spatial dynamics of range change supports the use of multiple datasets (e.g., gazetteers, fauna records, nature reserve scientific surveys, scientific research, and news) to contribute novel insights into our understanding of the dynamics of species responses to human pressure and climate change. In addition, they can be used to track range changes across longer timescales than those usually addressed in ecology or conservation biology. Our analyses combined multiple historical datasets with historical climate and species distribution models, which has not been attempted in previous research.

### 4.1 Model limitations

This study has several limitations. First, this research cannot provide completely accurate insight into the changes in sable distribution range over time. Due to our use of historical data, the results may contain sample biases resulting from data accessibility, spatial and temporal variables, and nonstandardized sampling (Turvey et al., 2015; Yang et al., 2016; Zhang et al., 2016). The predictors came from the surface daily climate dataset of 72 meteorological stations also contained sample biases resulting from spatial cluster (most of the meteorological stations next to the city) and data accuracy (the data precision increased after the 1990s). In addition, environmental niche models have limitations due to their representation of predictors. In this study, we excluded various nonclimatic factors, including those related to habitat and human impact. Furthermore, we were unable to access land coverage or accurate population data (in towns) over the timescale examined. Therefore, this model only contains predictors from climate and topography data. Although the occurrence points contain potential information on the excluded factors, the model results may still contain biases due to missing predictors. However, the model results are still enough to reveal such obvious range change through time.

### 4.2 Biogeographic characteristics over time

Based on our results, range change was particularly associated with two factors: climate and human influence. The range change was divergent in the three regions examined due to their complex terrains. Due to the lower human population density in the Greater Khingan Mountains since the 1950s, the sable population movement toward north mainly resulted from the poleward shifts under global warming. In the Lesser Khingan Mountains, the influence of the urbanization and climate change through time forced the sable population to shift the habitat to the north which tend to be colder and have the lower human population density. According to the landcover type in 2013 (Fig. S3 in Appendix S3), urban agglomeration and farmland can be the important threat to the sable population in the Lesser Khingan Mountains and Changbai Mountains. Therefore, our results partly demonstrate that these three regional sable populations were influenced by climate change, as well as by other factors (e.g., human population size), which is consistent with the results of Urban and Chen (Chen, Hill, Ohlemuller, Roy, & Thomas, 2011; Urban et al., 2013). This trend of range change would be continued in the near future. Moreover, we suggest that the trend of the human population size through time should be considered in species distribution modeling research.

### 4.3 Conservation implications

In responding to climate change, there is a particular need for climate change‐integrated conservation strategies (Butchart et al., 2010; Luo et al., 2015; Schloss et al., 2012; Willis et al., 2007). Many studies on this issue have suggested that climate influences local species abundance, community structure and biodiversity, phenology, and species range, but only a few have addressed their research using a historical perspective (Ban et al., 2014; Beaugrand, Edwards, Raybaud, Goberville, & Kirby, 2015; Davies et al., 2014; Mathur & Padalia, 2006; McClenachan et al., 2012; Rick & Lockwood, 2012; Saikia, Kalita, & Saikia, 2009). We suggest that conservation strategies based on historical information may provide a more realistic perspective. The sable population in Northeast China has divided into three regional populations in the Greater Khingan Mountains, Lesser Khingan Mountains, and Changbai Mountains. In the Greater Khingan Mountains, reducing human influences in the northern area may be the most effective management strategy, given that the sables’ elevation distribution appears to have decreased due to climate change and that the lower elevations are associated with higher human population densities (Figure 2; Fig. S2 in Appendix S3). Securing existing protected areas to maintain habitats, and establishing natural corridors in the northern region of the Lesser Khingan Mountains, might be suitable management strategies to protect this sable population (Figure 2; Fig. S2 in Appendix S3). The population in the Changbai Mountains may be facing the greatest challenge, as their migration to higher elevations in response to climate change limits space for their distribution. In addition, this region has experienced strong human influences for many years (Figure 2; Fig. S2 in Appendix S3). As the vulnerability of this population, due to climate change, may be higher than the other populations, active conservation strategies should be considered, including increasing connectivity, assisted migration, ex situ conservation, and reintroduction.

## Conflict of Interest

None declared.
