# Supplementary material for: Geographic characteristics of sable (Martes zibellina) distribution over time in Northeast China
Source: Ecol Evol. 2017 Apr 25;7(11):4016–23. doi: 10.1002/ece3.2983 (PMC5468152; doi:10.1002/ece3.2983)
Supplement: Supplementary file 1 [file ECE3-7-4016-s001.docx]

*Journal of Ecology and Evolution*

**SUPPORTING INFORMATION**

**Geographic characteristics of sable (***Martes zibellina***) distribution over time in Northeast China**

Rui Zhang^a^, Li Yang^a^, Lin Ai^a^, Qiuyuan Yang, Minhao Chen, Jingxi Li, Lei Yang, Xiaofeng Luan

**Appendix S1** The record data (*Martes zibellina*) of sable reference

Table S1. Important distribution information from other resources (all in Chinese)

| *Resource* | *Type* | *Year* | *Location* |
| --- | --- | --- | --- |
| Fauna(Wild animal of the southwest slope of Changbai Mountain) | specimen | 1980s | Changbai Korean Autonomous county, Fusong and Hunjiang, Jingyu, Huinan, Liuhe and Tonghua. |
| Fauna(Heilongjiang Beast) | investigation | 1980s | Great Khingan Mountains, Lesser Khingan Mountains and the eastern mountains |
| Fauna(Great Khingan Mountains Wildlife) | specimen | 1980s | Mohe, Tahe, Huzhong, Xinlin |
| Fauna(Rare and Endangered Species of Vertebrates of Northeast China) | investigation | 1990s | Northern of the Great Khingan Mountains, Lesser Khingan Mountains, Northern of the Zhangguangcai Mountains, Changbai Mountains, Southeast of the Wuchang county |
| Survey Report on the Northeast Mammalia | specimen | 1950s | Muling, Shangzh (Heilongjiang Province) Dunhua, Antu, Fusong, Changbai (JilinProvince) |
| Checklist of Mammal Specimens of Heilongjiang Province | specimen | 1970s | Hailin, Shuangcheng, Suifenhe, Bei'an |
| Survey Report of the State Forestry in Heilongjiang Province | investigation | 2000s | Dahailin, Dongjingcheng, Suiyang, Zhanhe, Tongbei, Suiling, Nancha, Dailing and Langxiang |
| Article | investigation | 1996 | Northern of the Great Khingan Mountains |
| Article | investigation | 2013 | Heilongjiang Cuibei Wetland Nature Reserve |
| Article | investigation | 2015 | Heilongjiang Lingfeng Nature Reserve |
| Article | investigation | 2001 | Heilongjiang Mergus squamatus Nature Reserve |
| Article | investigation | 2000 | A'bahe basin in the Great Khingan Mountains |
| News | Infrared camera | 2015 | Heilongjiang Zengfengling Nature Reserve |
| News | Witness | 2015 | Tumenjiangyuan National Forest Park |
| News | Witness | 2015 | The West Scenic of the Great Khingan Mountains |
| News | Witness | 2015 | Heilongjiang Taipinggou Nature Reserve |
| News | killed | 2015 | Tuanbei Forestry in the Huangnihe Linyeju |
| News | killed | 2015 | Haiyuan Forestry |
| News | Witness | 2015 | Tuqiang Linyeju |

**Appendix S2** Selection of variables

A subset of predictor variables were selected from the 22 eco-geographic variables (Table S3). To reduce over-fitting for the output per periods, we selected the predictors using correlation analysis. We built Pearson correlation matrices for the bio-climate and topography datasets. We drew a network diagram using a raster structure using the igraph package in R software to facilitate variable selection (Hijmans 2014; Csardi & Nepusz 2006). In the network diagram, highly correlated variables (|r| ≥ 0.8) were linked by lines and clustered together (Fig S1). Under the assumption that extreme climate index variables (e.g., BIO5, BIO6, BIO14, etc.) represented limiting factors for the species, we prioritised those variables within each variable cluster. 11 - 14 environmental variables were selected. For all coefficients ≥ 0.8, we removed the variable with the lower value in the percentage contribution (from the MaxEnt) in the pair (Table S3). Selected variables used in the species distribution models of sable in the Northeast China for different periods are shown in Table S1.


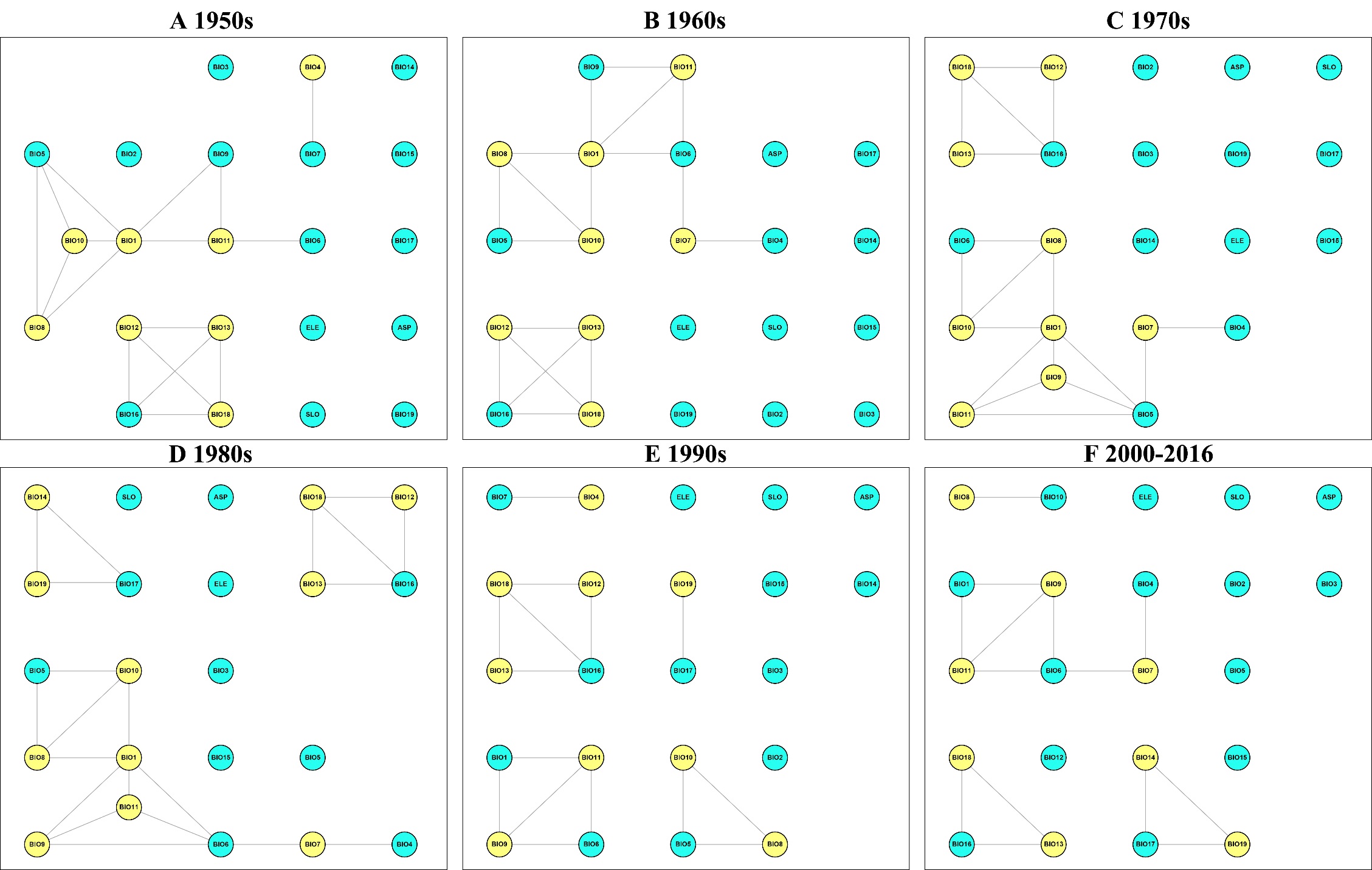


Fig S1. Network diagram of 23 environmental variables. Two circles are linked if the two represented variables are highly correlated (|r|≥ 0.8). The variables selected are marked blue.

Table S2. Eco-geographic variables used in species distribution models of sable in Northeast China for different periods.

| Variables | Describe | Code | 1950s | 1960s | 1970s | 1980s | 1990s | 2000s |
| --- | --- | --- | --- | --- | --- | --- | --- | --- |
| BIO1 | Annual Mean Temperature | BIO1 |  |  |  |  | ● | ● |
| BIO2 | Mean Diurnal Range (Mean of monthly (max temp - min temp)) | BIO2 | ● | ● | ● | ● | ● | ● |
| BIO3 | Isothermality (BIO2/BIO7) (* 100) | BIO3 | ● | ● | ● | ● | ● | ● |
| BIO4 | Temperature Seasonality (standard deviation *100) | BIO4 |  | ● | ● | ● |  | ● |
| BIO5 | Max Temperature of Warmest Month | BIO5 | ● | ● | ● | ● | ● | ● |
| BIO6 | Min Temperature of Coldest Month | BIO6 | ● | ● | ● | ● | ● | ● |
| BIO7 | Temperature Annual Range (BIO5-BIO6) | BIO7 | ● |  |  |  | ● |  |
| BIO8 | Mean Temperature of Wettest Quarter | BIO8 |  |  |  |  |  |  |
| BIO9 | Mean Temperature of Driest Quarter | BIO9 | ● | ● |  |  |  |  |
| BIO10 | Mean Temperature of Warmest Quarter | BIO10 |  |  |  |  |  | ● |
| BIO11 | Mean Temperature of Coldest Quarter | BIO11 |  |  |  |  |  |  |
| BIO12 | Annual Precipitation | BIO12 |  |  |  |  |  | ● |
| BIO13 | Precipitation of Wettest Month | BIO13 |  |  |  |  |  |  |
| BIO14 | Precipitation of Driest Month | BIO14 | ● | ● | ● |  | ● |  |
| BIO15 | Precipitation Seasonality (Coefficient of Variation) | BIO15 | ● | ● | ● | ● | ● | ● |
| BIO16 | Precipitation of Wettest Quarter | BIO16 | ● | ● | ● | ● | ● | ● |
| BIO17 | Precipitation of Driest Quarter | BIO17 | ● | ● | ● | ● | ● | ● |
| BIO18 | Precipitation of Warmest Quarter | BIO18 |  |  |  |  |  |  |
| BIO19 | Precipitation of Coldest Quarter | BIO19 | ● | ● | ● |  |  |  |
| Elevation |  | ELE | ● | ● | ● | ● | ● | ● |
| Aspect |  | ASP | ● | ● | ● | ● | ● | ● |
| Slope |  | SLO | ● | ● | ● | ● | ● | ● |

Note: Topography was calculated from SRTM 90 m Digital Elevation Data (http://srtm.csi.cgiar.org/) (Jarvis et al., 2008); The variables selected are marked black dot.

**REFERENCES**

Jarvis, A., H.I. Reuter, A. Nelson, E. Guevara. (2008) Hole-filled SRTM for the globe Version 4, available from the CGIAR-CSI SRTM 90 m Database (http://srtm.csi.cgiar.org)

**Appendix S3** The sable distribution range change and human influence

Table S3 Range sizes and five factors in different period

| Periods | Range  (km^2^) | Human population size (million) | Annual Mean Temperature (Bio1) (℃) | Max Temperature of Warmest Month (Bio5) (℃) | Min Temperature of Coldest Month (Bio6) (℃) | Annual Precipitation (Bio12) (mm) |
| --- | --- | --- | --- | --- | --- | --- |
| 1950s | 631522 | 27.182 | 0.442 | 29.987 | -43.869 | 29838.125 |
| 1960s | 607354 | 40.094 | 0.292 | 30.124 | -47.596 | 49690.269 |
| 1970s | 532024 | 56.486 | 0.544 | 29.883 | -41.083 | 44631.515 |
| 1980s | 447252 | 61.356 | 0.914 | 29.341 | -40.897 | 55276.771 |
| 1990s | 305706 | 73.887 | 2.348 | 38.369 | -39.748 | 53694.859 |
| 2000-2016 | 304932 | 75.664 | 2.339 | 42.903 | -39.458 | 43524.519 |

Note: The range for each periods were assessed by summing the numbers of the respective related pixels. The human population size data in this region was collected from the 1st-6th national population census and some new gazetteers ([Statistics, 1984](#_ENREF_6); [Song, 1987](#_ENREF_4); [Cheng & Zhang, 1992](#_ENREF_2); [Chen & Shi, 2000](#_ENREF_1); [Zhang, 2004](#_ENREF_8); [Zhu, 2005](#_ENREF_9); [She & Xiong, 2010](#_ENREF_3); [Statistical & Jilin, 2010](#_ENREF_5); [Xu & Wan, 2010](#_ENREF_7)); and the region in Fig S2 was based on human population size region (Statistics, 1984; [Song, 1987](#_ENREF_4)) (Fig S2). Bio1, Bio5, Bio6, and Bio12 were calculated by ANUSPLIN v4.36.

**REFERENCES**

Chen, Y.Y. & Shi, X.Z. (2000) *Inner Mongolia Statistical Yearbook (2000)(in Chinese)*. China Statistics Press, Beijing.

Cheng, Z. & Zhang, Z.X. (1992) *Jilin Shengzhi(in Chinese)*. Jilin People's Publishing House, Changchun.

She, J.X. & Xiong, W. (2010) *Heilongjiang Statistical Yearbook (2010) (in Chinese)*. China Statistics Press, Beijing.

Song, Q.G. (1987) *China's population - Inner Mongolia(in Chinese)*. China Financial & Economic Publishing House, Beijing.

Statistical, J.B.o. & Jilin, S.O.o.t.N.B.o.S.i. (2010) *Jlin Statistical Yearbook (2010) (in Chinese)*. China Statistics Press, Beijing.

Statistics, H.B.o. (1984) *Development of Heilongjiang Province (1949-1983(in Chinese))*. Heilongjiang Bureau of Statistics, Harbin.

Xu, Q.W. & Wan, L.Q. (2010) *Inner Mongolia Statistical Yearbook (2010) (in Chinese)*. China Statistics Press, Beijing.

Zhang, S.W. (2004) *China's Population at the Turn of the Centuries - Heilongjiang Province(in Chinese).* China Statistics Press, Beijing.

Zhu, X.D. (2005) *China's Population at the Turn of the Centuries - Jilin Province(in Chinese)*. China Statistics Press, Beijing.

Table S4 Range sizes and five factors in different region

| Region | Decade | Range (km^2^) | Longitude (°) | Latitude (°) | Elevation (m) |
| --- | --- | --- | --- | --- | --- |
| Greater Khingan Mountains | 1950s | 84250 | 122.563 | 50.641 | 705.822 |
|  | 1960s | 77863 | 122.429 | 50.743 | 710.440 |
|  | 1970s | 67786 | 122.611 | 50.798 | 686.039 |
|  | 1980s | 72762 | 122.836 | 51.306 | 680.696 |
|  | 1990s | 44173 | 122.839 | 52.054 | 642.272 |
|  | 2000s | 45539 | 122.608 | 51.972 | 673.456 |
| Lesser Khingan Mountains | 1950s | 50465 | 127.865 | 49.099 | 337.106 |
|  | 1960s | 53558 | 127.737 | 48.778 | 354.719 |
|  | 1970s | 26424 | 126.950 | 49.642 | 395.083 |
|  | 1980s | 17149 | 127.270 | 49.423 | 406.247 |
|  | 1990s | 22554 | 127.708 | 49.340 | 407.294 |
|  | 2000s | 13522 | 128.262 | 49.548 | 361.013 |
| Changbai Mountains | 1950s | 148526 | 129.344 | 44.080 | 449.066 |
|  | 1960s | 142381 | 128.588 | 43.493 | 554.666 |
|  | 1970s | 145708 | 128.403 | 43.129 | 639.654 |
|  | 1980s | 111199 | 128.420 | 43.198 | 615.198 |
|  | 1990s | 69832 | 128.545 | 43.123 | 691.292 |
|  | 2000s | 78535 | 128.217 | 42.727 | 720.470 |

*Note:* Three region only contain the core area of the Mountains. Therefore, we exclude the plains for this analysis. The longitude and latitude were obtained from the range centroids. The elevation were obtained from potential distribution


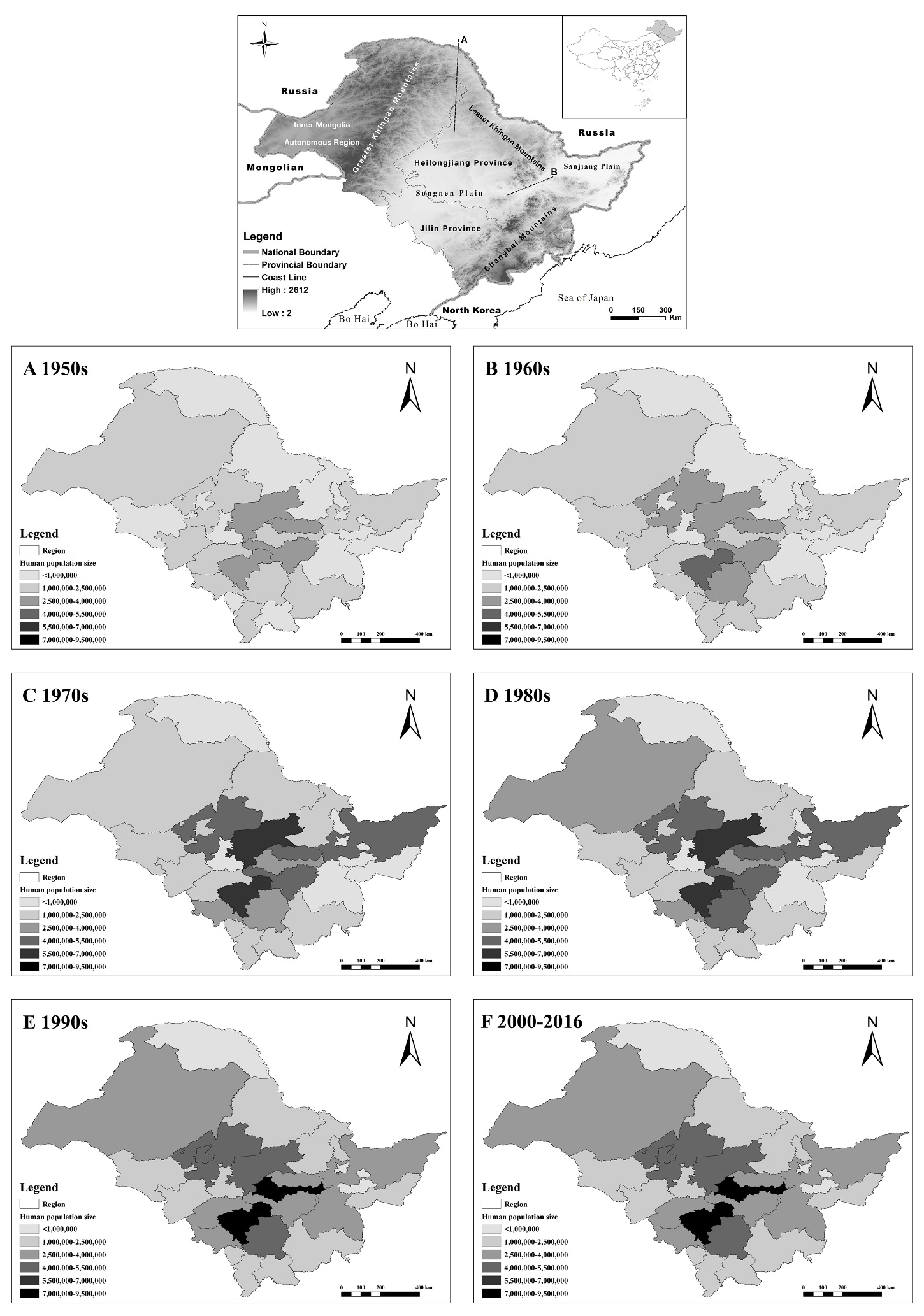


Fig S2. Human population size for different regions in different periods


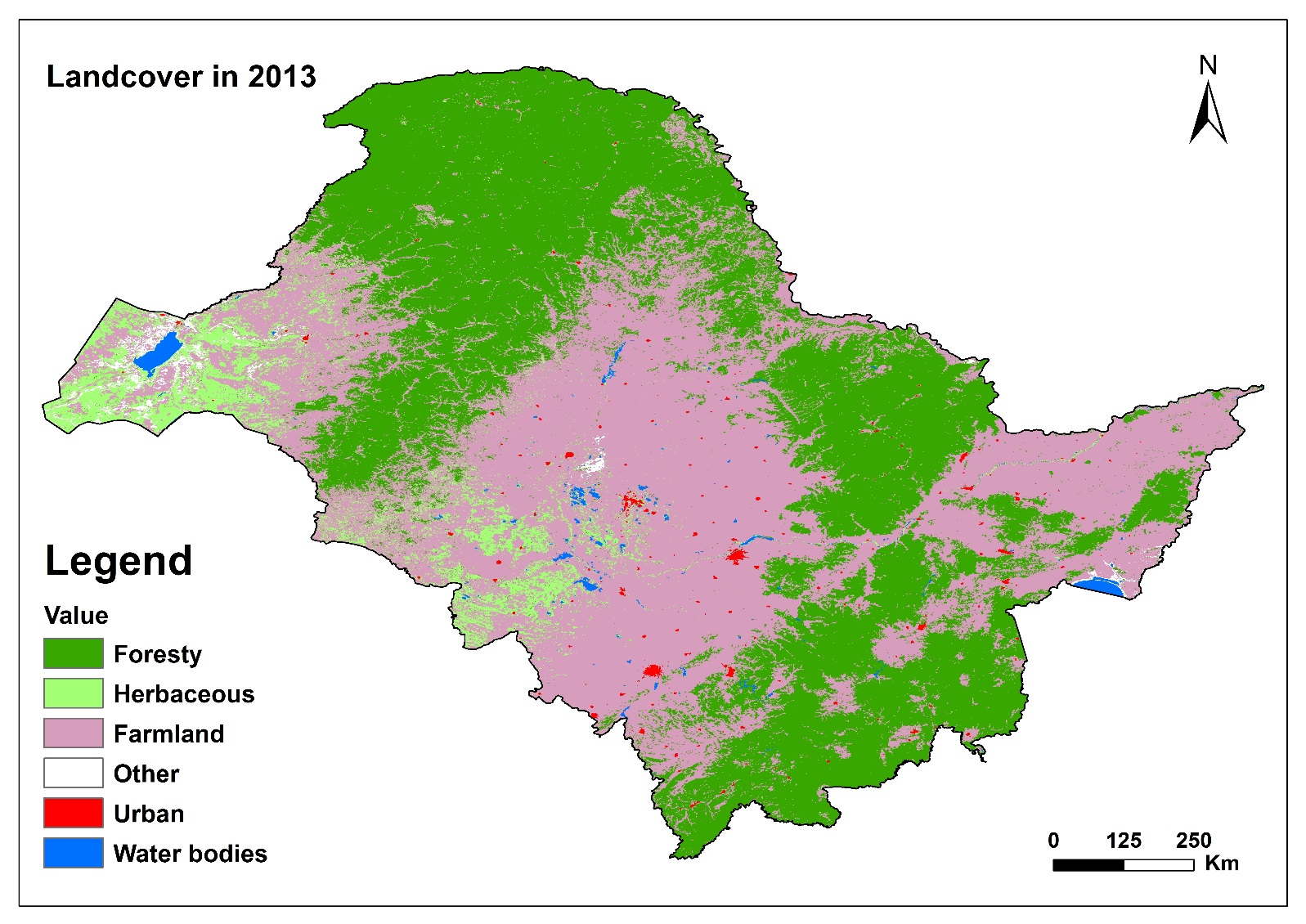


Fig S3. Landcover type in 2013 from The Global Land Cover by National Mapping Organizations (GLCNMO)

**REFERENCES**

TATEISHI, R., HOAN, N. T., KOBAYASHI, T., ALSAAIDEH, B., TANA, G. & PHONG, D. X. 2014. Production of Global Land Cover Data – GLCNMO2008. *Journal of Geography and Geology,* 6. doi: 10.5539/jgg.v6n3p99
